# The Potential Advantages of Tai Chi Chuan in Promoting Inhibitory Control and Spontaneous Neural Activity in Young Adults

**DOI:** 10.3389/fnbeh.2021.747733

**Published:** 2021-11-04

**Authors:** Qi-Qi Shen, Heng-Chan Yin, Lei Cui, Jing-Yi Zhang, Dong-Ling Wang, Li-Na Zhu, Yuan Wang, Xiu-Juan Li

**Affiliations:** ^1^College of P. E. and Sports, Beijing Normal University, Beijing, China; ^2^State Key Laboratory of Cognitive Neuroscience and Learning, Beijing Normal University, Beijing, China; ^3^PE Department, Renmin University of China, Beijing, China

**Keywords:** Tai Chi Chuan, exercise intervention, spontaneous neural activity, brain plasticity, functional magnetic resonance imaging, inhibition control

## Abstract

Tai Chi Chuan (TCC) is assumed to exert beneficial effects on functional brain activity and cognitive function in elders. Until now, empirical evidence of TCC induced intra-regional spontaneous neural activity and inhibitory control remains inconclusive. Whether the effect of TCC is better than that of other aerobic exercises is still unknown, and the role of TCC in younger adults is not yet fully understood. Here we used resting-state functional MRI (fMRI) to investigate the effects of 8-week TCC (*n* = 12) and brisk walking (BW, *n* = 12) on inhibitory control and fractional amplitude of low-frequency fluctuations (fALFF). The results found that TCC had significant effects on inhibitory control performance and spontaneous neural activity that were associated with significantly increased fALFF in the left medial superior frontal gyrus (*Cohen’s d* = 1.533) and the right fusiform gyrus (*Cohen’s d* = 1.436) and decreased fALFF in the right dorsolateral superior frontal gyrus (*Cohen’s d* = 1.405) and the right paracentral lobule (*Cohen’s d* = 1.132).TCC exhibited stronger effects on spontaneous neural activity than the BW condition, as reflected in significantly increased fALFF in the left medial superior frontal gyrus (*Cohen’s d* = 0.862). There was a significant positive correlation between the increase in fALFF in the left medial superior frontal gyrus and the enhancement in inhibitory control performance. The change in fALFF in the left medial superior frontal gyrus was able to explain the change in inhibitory control performance induced by TCC. In conclusion, our results indicated that 8 weeks of TCC intervention could improve processing efficiency related to inhibitory control and alter spontaneous neural activity in young adults, and TCC had potential advantages over BW intervention for optimizing spontaneous neural activity.

## Introduction

Inhibition is a cognitive control process that allows us to suppress dominant and automatic responses to goal-irrelevant stimuli when needed (Nigg, 2000). It plays significant and intricate roles in different dimensions of thinking and behavioral processes, including attention (Gardner and Long, 1962), emotional perception (Ozonoff et al., 1991), and emotional regulation (Pessoa, 2009; Hendricks and Buchanan, 2016). Intervention studies have demonstrated the plasticity of inhibition function. Over the years, substantial evidence has suggested that physical exercise, an inexpensive and relatively safe intervention, is an effective method for enhancing inhibitory control. Prior studies have reported substantial exercise effects on tasks that measure inhibitory control (Tomporowski et al., 2008; van Uffelen et al., 2008; Smith et al., 2010; Chaddock et al., 2012; Verburgh et al., 2014; Tang et al., 2017; Li et al., 2020; Song et al., 2020) with increased task-related brain activation (Krafft et al., 2014; e.g., dorsolateral prefrontal cortex, medial frontal gyrus, superior frontal gyrus, middle frontal gyrus, superior temporal gyrus, cingulate gyrus, and insula). However, even in the absence of any clear sensory input or behavioral output, the brain remains active (Fox and Raichle, 2007). Spontaneous fluctuations of the blood oxygen level-dependent (BOLD) signals in functional magnetic resonance imaging (fMRI) are important manifestations of spontaneous neural activity. Recently, an increasing number of studies have used resting-state fMRI to explore spontaneous neural activity (Amad et al., 2017; Flodin et al., 2017). Furthermore, motor learning and exercise interventions have been found to induce neural plasticity in the resting brain (Wayne and Fuerst, 2013).

Tai Chi Chuan (TCC), an Asian mindfulness exercise for integrating body and mind (Wayne and Fuerst, 2013), has been shown to improve cognitive functions, such as memory and attention (Chan et al., 2008; Tao et al., 2017c). In addition, resting-state fMRI methods, like voxel-mirrored homotopic connectivity (VMHC; Chen et al., 2020), regional homogeneity (ReHo; Wei et al., 2014), and resting-state functional connectivity (rsFC; Tao et al., 2017a), have confirmed that TCC can alter spontaneous neural activity in elderly individuals. Most of these studies have described the critical functional connectivity or functional integration between different brain areas related to cognitive functions, but they couldn’t provide direct information on the amplitude of brain activity in each brain region. Spontaneous low-frequency (0.01–0.08 Hz) fluctuations (LFF) have been linked to spontaneous neural activity in a region. LFF closely resembles the task-dependent activation pattern in fMRI (Biswal et al., 1995), thus providing valuable characteristic information of the spontaneous neural activity (Goldman et al., 2002; Lu et al., 2007; Mantini et al., 2007). The amplitude of the LFF (ALFF) has been used as a reliable and sensitive indicator of spontaneous neural activities in many studies (Guo et al., 2013). It has also been shown that resting-state ALFF could be used as the marker for intervention-induced neural plasticity in elderly subjects (Yin et al., 2014). However, the ALFF method is also sensitive to physiological noise. Zou et al. (2008) have proposed a fractional ALFF (fALFF) approach, which determines the ratio of the low-frequency power spectrum to that of the entire frequency range to avoid the background effects of physiological noise. Studies have revealed that correlation analysis between the fALFF and behavioral characteristics could be valuable gauges for examining the potential neural activity underlying cognitive control (Mennes et al., 2011; Deng et al., 2016). Recently, several studies have explored the effect of TCC on fALFF in older adults. Tao et al. (2017b) found that TCC and Baduanjin (popular mind-body practices) interventions can modulate medial prefrontal cortex (mPFC) fALFF and promote memory function and a significant correlation between mPFC fALFF changes and memory. Wei et al. (2017) have demonstrated that TCC practitioners have lower fALFF in the left-lateralized frontoparietal region than that in the controls (those with no regular TCC practice). However, the group difference in inhibitory control was not significant. In fact, the changes in spontaneous brain activity in older adults who practiced Tai Chi for a long period of time were not related to the performance of inhibitory control tasks. What about young adults? Since the causal relationship could not be established in a cross-sectional study, the potential role of TCC-induced intra-regional spontaneous neural activity on inhibitory control is still unknown. Besides, whether TCC, a mind-body exercise that includes mindfulness, has a different effect on inhibitory control and spontaneous neural activity from general aerobic exercise (non-mindfulness) is also worth further discussion, and the role of TCC in younger adults is not fully understood.

Therefore, the present study aimed to determine the potential advantages of TCC for promoting inhibitory control and modulating spontaneous neural activity in young adults. We hypothesized that: (1) a long-term TCC intervention could induce changes in the inhibitory control and fALFF in the brain; (2) the effect of TCC might be stronger than that of other aerobic exercises (brisk walking); and (3) inhibitory control changes induced by TCC might be associated with changes in fALFF.

## Materials and Methods

### Participants and Study Design

The institutional review board of the National Key Laboratory of Cognitive Neuroscience and Learning approved the experimental procedures. All procedures were conducted in compliance with the Declaration of Helsinki. Forty-two college students were recruited by advertisement, of which six were excluded because either they had metal implants in their body or were in the abnormal range of depression scale or did not fit with the timing of the exercise intervention. Thirty-six healthy young adults without regular exercise habits were randomly divided into three groups matched by sex, namely the TCC intervention group, the BW group, and the control group. Participants were assessed by the resting-state fMRI scan and inhibitory control test before and after the 8-week intervention period. All participants provided written informed consent and were compensated for their participation. All participants completed the experiment.

### Exercise Intervention Procedures

The exercise group participated in three weekly sessions for group training for 8 weeks in gym (Cui et al., 2019). The TCC group received Bafa Wubu of Tai Chi exercise. Bafa Wubu of Tai Chi has been systematically refined and organized by the General Administration of Sports of China based on the existing 24-form Tai Chi consisting of a set of Tai Chi routines popularly characterized by culture, fitness, and simplicity (Flodin et al., 2017). The first 1–3 week is the stage for “Building Xing,” when the subject will master all the movements skillfully. The next 4–6 weeks are for “Conveying Qi,” which focuses on the respiration of the subject during the intervention. And the final 7–8 week is mainly the stage of “using Yi,” which emphasizes use mind to guide the movements and finally achieves the harmony of Xing, Qi, and Yi. The BW group received three weekly sessions for group training of BW exercise in gym (open space). Each training session (TCC and BW) lasted 60 min, starting with a 5 min warming-up and ending with a 5 min cooling-down. The polar watch (PolarElectro Oy, Kempele, Finland) was used to monitor participants’ heart rates during the exercise sessions to ensure moderate exercise intensity (64% to 76% HRmax). The control group routinely maintained their original daily and physical activity habits and was instructed not to engage in any additional exercises.

### Data Acquisition

#### Inhibitory Control Assessment

For each participant, we applied a modified flanker task to measure inhibitory control before and after the intervention period. The main aspect assessed in the flanker task is interference control at an attention level, which requires suppressing distracting stimuli and competing for response tendencies (Van den Bussche et al., 2020). Each measurement was performed after the resting-state fMRI scan outside the scanner and collected with E-prime software^1^.

The stimuli were comprised of two conditions (Uono et al., 2017; Hintz et al., 2020): congruent and incongruent. Each condition was presented in 60 trials, for a total of 120 trials. Under congruent conditions, the distractor arrows were pointed in the same direction as the target arrow (<<<<< and >>>>>), while under incongruent conditions, the arrows pointed in opposite directions (<<><< and >><>>). During each trial, the participants first saw a fixation cross for 500 ms, and then the target stimulus was presented for 1,000 ms (Zhu et al., 2021). Participants were asked to respond as quickly and accurately as possible by pressing the button according to the direction of a centrally presented arrow that appeared on the computer screen and were asked to perform 12 practice trials and then complete two blocks of 60 trials each. The congruent and incongruent trials were presented in random order with equal probability in each block. Flanker accuracy was evaluated using mean accuracy, and speed was evaluated using mean Response Time (RT). The performance indicator (mean RT to incongruent trials minus mean RT to congruent trials) was used to measure reflected the ability to inhibit task-irrelevant distractor information (Hintz et al., 2020).

To minimize the learning effects, the participants performed a training task before the first study day, and the participants were asked to complete a training test until they reached 85% accuracy on the practiced flanker test.

#### MRI Data Acquisition

For each participant, we obtained an 8 min resting-state fMRI scan before and after the 8-week intervention period. All participants were asked to refrain from intense physical activity, caffeine, and alcohol consumption for 24 h before the day of testing. Data were acquired using a 3.0T MRI system (Siemens Magnetom Prisma; Erlangen, Germany) with a 64-channel head coil, which was located in the Beijing Normal University Imaging Center for Brain Research. Functional images were acquired using an echo-planar sequence sensitive to blood oxygenation level-dependent contrast (Xu et al., 2016; Cui et al., 2019, 2021): TR = 2,000 ms, TE = 30 ms; FA = 90°, slice thickness = 3.5 mm, 33 axial slices, voxel size = 3.5 × 3.5 × 3.5 mm, FOV = 224 × 224 mm, 240 volumes. The participants were instructed to keep their eyes open without falling asleep and to move as little as possible. As assessed by a questionnaire, none of the subjects reported falling asleep during the scanning or being uncomfortable during or after the procedure. A high resolution three-dimensional T1-weighted magnetization-prepared rapid gradient-echo images were acquired (Grewe et al., 2015; Wei et al., 2017): TR = 2,530 ms, TE = 2.98 ms, inversion time = 1,100 ms, FA = 7°, slice thickness = 1 mm, 192 sagittal slices, voxel size = 0.5 × 0.5 × 1 mm, FOV = 224 × 256 mm.

### Data Analysis

#### Inhibitory Control Analysis

Behavioral analysis was performed using SPSS (International Business Machines Corp., NY, USA). Repeated-measures ANOVA was first conducted to explore changes in task performance in the TCC, BW, and control groups from pre-intervention to post-intervention. When the interaction effect was significant, the *post hoc* test was subsequently analyzed. For multiple comparisons of changes (post minus pre-test) of each dependent variable, the one-way ANOVA test followed by the Bonferroni *post hoc* test was used. By treating the changes in inhibitory control from before to after the exercise intervention as the dependent variable, the differences in each dependent variable among the TCC BW, and control groups were compared. All the tests were performed 2-sided, and *p* ≤ 0.05 was considered significant in this analysis.

#### Resting-State fMRI Image Pre-processing

Functional imaging data pre-processing was implemented using GRETNA toolbox^2^ based on the SPM software^3^, including the following conventional steps (Zou et al., 2008; Xu et al., 2016; Cui et al., 2021): (1) discarding the first 10 time points, allowing for signal equilibrium and adaptation of the participants to the scanning noise; (2) compensating for systematic slice-dependent time shifts; (3) correcting for head movement with rigid body translation and rotation parameters; (4) normalizing into Montreal Neurological Institute (MNI) space using unified segmentation on T1-weighted images and re-slicing into 3-mm cubic voxels; (5) spatial smoothing with a 4-mm full-width at half-maximum Gaussian kernel; (6) removing the linear trend signal; and (7) regressing out nuisance variables, including head motion parameters (Friston-24), by averaging the white matter (WM) signal from the deep cerebral WM and the cerebrospinal fluid (CSF) signal-averaged from the ventricles to reduce non-neuronal contributions further. All the data used in this study satisfied the criteria for spatial movement in any direction (x, y or z) <2 mm translation or 2° rotation.

#### Spontaneous Neural Activity Analysis

The REST toolbox^4^ (*Q* = rest; Qiu et al., 2018) was used to perform fALFF measurements. Following the pre-processing, the rs-fMRI time series for each voxel was transformed into the frequency domain using the fast Fourier transform. In each voxel of the brain, the fALFF was computed as the ratio between the total amplitudes across 0.01–0.08 Hz and the sum of amplitudes across the entire frequency domain (0–0.25 Hz). Then, bandpass (0.01–0.08 Hz) filtering was carried out to reduce the influence of physiological noise. For each fALFF value, we converted the normalized expression values to Z scores to eliminate the differences of whole-brain fALFF in the overall level between individuals. A 3-group (TCC, BW, and control) × 2-time (pre and post-test) repeated measures ANOVA was performed on the whole brain fALFF to obtain the brain regions with group × time significant interaction effects (FWE corrected *p* ≤ 0.05 with a cluster size ≥5) using SPM. The xjView toolbox^5^ was used to mask the four significant brain regions. Then, the REST toolbox was used to extract the fALFF values from the four brain regions of each subject, followed by *post hoc* statistical analysis in SPSS 25.0.

#### Association Between Inhibitory Control and Spontaneous Neural Activity Changes

Pearson correlation and regression analysis were used to estimate the relationship between changes in fALFF (post minus pre-test) and changes in inhibitory control (post minus pre-test) with TCC training.

## Results

### Demographic Characteristics

Demographic characteristics are listed in Table 1. There were no significant differences among these three groups.

**Table 1:** Demographic characteristics.

Items	Tai Chi Chuan M (SD)	Brisk walking M (SD)	Control M (SD)	*F*	*p*
Sex (Male/Female)	2/10	2/10	2/10	—	—
Age (years)	21.83 (2.48)	21.92 (2.28)	21.75 (2.45)	0.014	0.986
Handedness (Left/Right)	0/12	0/12	0/12	—	—
Education (years)	16.33 (2.23)	16.41 (2.27)	16.33 (1.50)	0.007	0.993
BMI (kg/m^2^)	20.21 (2.54)	19.15 (2.06)	21.71 (2.96)	3.07	0.06

### Inhibitory Control Results

For the accuracy, there were no significant main effects of group and time, and no significant interaction of group by time was found in both the congruent and incongruent conditions (*p*s < 0.05). The means and standard deviations (SD) are listed in Table 2.

**Table 2:** Flanker task performance.

	Tai Chi Chuan M(SD)	Brisk walking M(SD)	Control M(SD)
*Pre*
Inhibitory control RT (ms)	116.82 (16.63)	121.09 (30.36)	119.03 (31.36)
Congruent accuracy (%)	0.969 (0.040)	0.963 (0.042)	0.957 (0.037)
Incongruent accuracy (%)	0.956 (0.025)	0.962 (0.039)	0.953 (0.015)
*Post*			
Inhibitory control RT (ms)	95.66 (21.80)	115.32 (32.24)	127.23 (33.65)
Congruent accuracy (%)	0.967 (0.039)	0.962 (0.043)	0.953 (0.035)
Incongruent accuracy (%)	0.954 (0.021)	0.959 (0.031)	0.955 (0.015)

**Figure 1:**
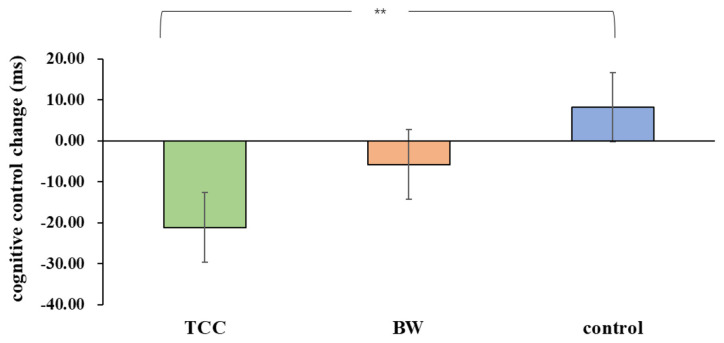
Group differences in inhibitory control task reaction times (RT). **p ≤ 0.01.

For the inhibitory control RT, there was a significant group × time interaction (*F*_(1,33)_ = 3.903, *p* = 0.03, $\eta_{\text{p}}^{2}$ = 0.191) on the inhibitory control performance. There was no significant main effect of the group (*F*_(1,33)_ = 3.903, *p* = 0.257, $\eta_{\text{p}}^{2}$ = 0.079) or time (*F*_(1,33)_ = 2.115, *p* = 0.155, $\eta_{\text{p}}^{2}$ = 0.060) on the inhibitory control. A follow-up analysis deconstructing the interaction effects revealed that there was a significant difference between pre- and post-test in the TCC group (*F*_(1,33)_ = 8.10, *p* = 0.008, *Cohen’s d* = 0.611), but no significant differences were observed between those either in the BW group (*F*_(1,33)_ = 0.60, *p* = 0.443, *Cohen’s d* = 0.326) or in the control group(*F*_(1,33)_ = 1.22, *p* = 0.278, *Cohen’s d* = 0.376). Moreover, there was no significant difference among three groups in the pre-test (*F*_(1,33)_ = 0.08, *p* = 0.928, ($\eta_{\text{p}}^{2}$ = 0.005) but was in the post-test(*F*_(1,33)_ = 3.46, *p* = 0.043, ($\eta_{\text{p}}^{2}$ = 0.173). The inhibitory control performance changes (post minus pre-test) were compared among the three groups. The results showed that inhibitory control performance was significantly improved in the TCC group compared to that in the control group (*p* = 0.009, *Cohen’s d* = 1.014). The other differences did not produce any statistical significance (*ps* > 0.05; Figure 1).

Next, we conducted a *post hoc* power analysis using the software G*Power (version 3.1.9.7; Kiel University, Kiel, Germany) to confirm the sample sizes. We used a power analysis with Effect size *f* = 0.485 ($\eta_{\text{p}}^{2}$ = 0.191), *α* error of probability = 0.05, Number of groups = 3, Number of measurements = 2, and correlation = 0.554. Within our chosen total sample size, the power (1-*β*) was approximately 0.99.

### fALFF Results

**Figure 2:**
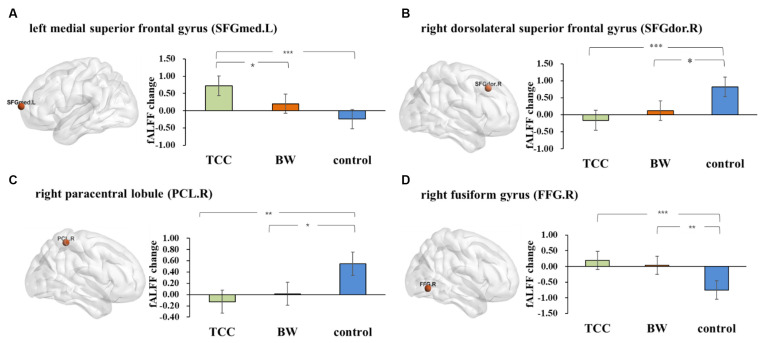
The fractional amplitude of low-frequency fluctuations (fALFF) results. Changes in the fALFF after 8 weeks of Tai Chi Chuan (TCC) and BW. **(A)** *Post hoc* statistical analysis results of fALFF changes in the SFGmed.L-left medial superior frontal gyrus. **(B)** *Post hoc* statistical analysis of the fALFF changes in SFGdor.R-right dorsolateral superior frontal gyrus. **(C)** *Post hoc* statistical analysis results of the fALFF changes in the PCL.R-right paracentral lobule. **(D)** *Post hoc* statistical analysis results of the fALFF changes in the FFG.R-right fusiform gyrus. *p ≤ 0.05, **p ≤ 0.01, and ***p ≤ 0.001.

Repeated measures ANOVA on the fALFF values yielded four significant interactions (Figure 2): the left medial superior frontal gyrus (SFGmed.L; cluster size: 5; peak MNI coordinates: −12, 69, 9; *F* = 55.33), right dorsolateral superior frontal gyrus (SFGdor.R; cluster size: 7; peak MNI coordinates: 18, 27, 42; *F* = 46.37), right paracentral lobule (PCL.R; cluster size: 6; peak MNI coordinates: 3, −42, 60; *F* = 50.56), and right fusiform gyrus (FFG.R; cluster size: 8; peak MNI coordinates: 30, −66, −6; *F* = 54.63).

The fALFF changes (post minus pretest) were compared among the three groups. The results showed that there was a significant increase in the SFGmed.L (*p* < 0.001, *Cohen’s d* = 1.533) and FFG.R (*p* = 0.001, *Cohen’s d* = 1.436) and a significant decrease in the SFGdor.R (*p* = 0.001, *Cohen’s d* = 1.405) and PCL.R (*p* = 0.003, *Cohen’s d* = 1.132) in the TCC group compared to that of the control group. There was a significant increase in the FFG.R (*p* = 0.006, *Cohen’s d* = 1.398), and the increasing trend gradually slowed down in the PCL.R (*p* = 0.016, *Cohen’s d* = 1.374) and SFGdor.R (*p* = 0.018, *Cohen’s d* = 0.889) in the BW group compared to that of the control group. We also found a significant increase in the SFGmed.L (*p =* 0.041, *Cohen’s d* = 0.862) in the TCC group compared to that in the BW group.

Then, we conducted a *post hoc* power analysis. We used a power analysis with Effect size *f* = 0.588–0.692 ($\eta_{\text{p}}^{2}$ = 0.284–0.324), *α* error of probability = 0.05, Number of groups = 3, Number of measurements = 2. Within our chosen total sample size, the power (1-*β*) was approximately 0.99.

### Association Between Inhibitory Control and Spontaneous Neural Activity Changes

**Figure 3:**
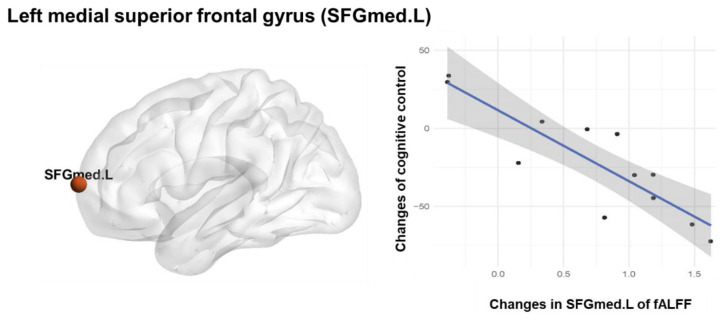
Association between Inhibitory control and spontaneous neural activity. Pearson correlation and regression analysis of fALFF changes and inhibitory control changes in the TCC group.

Significant correlation between the SFGmed.L fALFF changes and the inhibitory control performance changes (*r* = 0.871, *p* < 0.001 significant after Bonferroni correction; Figure 3) was observed in the TCC group. There was no significant correlation between the fALFF changes and the inhibitory control performance changes in the BW and control groups. A linear regression analysis was performed using the inhibitory control performance changes as a dependent variable and the SFGmed.L fALFF changes as an independent variable, which showed that TCC induced fALFF changes in the SFGmed.L could explain 73.5% (adjusted *R*^2^ = 0.735) of the observed variation in the inhibitory control performance improvement (*F*_(1,11)_ = 31.564, *β* = −0.871, *p* < 0.001; Table 3).

**Table 3:** fALFF changes with inhibitory control RT changes.

Independent variable	β	adjusted *R*^2^	*F*	95% CI	
				Lower	Upper
SFGmed.L fALFF changes	−0.871	0.735	31.564	−63.38	−27.4

## Discussion

This study aimed to examine the potential advantages of TCC for promoting inhibitory control and modulating spontaneous neural activity in young adults. To the best of our knowledge, this study was the first exercise intervention study to compare the impacts of TCC and general aerobic exercise on spontaneous neural activity and inhibitory control in young adults. We found that TCC exercise significantly increased inhibitory control performance and induced spontaneous neural activity changes. While BW exercise had no significant effect on the inhibitory control. Importantly, TCC had comparatively stronger effects on spontaneous neural activity than BW exercise, as indicated by significantly increased fALFF in SFGmed.L. In addition, there was a significant positive correlation between the enhancement in inhibitory control performance and the increase in fALFF in the SFGmed.L.

### Tai Chi Chuan Promoted Inhibitory Control Performance

Our findings indicated that an 8-week TCC intervention could improve inhibitory control, which was consistent with previous studies on mind-body exercise. Other studies have also found that mind-body exercises such as TCC can improve inhibitory control (Wayne et al., 2014; Zhang et al., 2018). Inhibitory control allows us to suppress dominant and automatic responses to goal-irrelevant stimuli. TCC may improve inhibitory control in the following respects.

From the perspective of movement types, TCC requires participants to abide by the basic principle of mind-body relaxation. On this basis, under the guidance of the mind, combined with breathing, the movements are correctly completed in a concentrated manner (Shaojun, 2019). During the entire movement process, individuals were asked to be intently guided by their minds and continue to be aware of their movements and respiratory rhythms so that their attention could be continuously improved (Cui et al., 2019, 2021), with better and timely modifications of the information related to inhibitory control depending on the needs of the task, which resulted in improved inhibitory control. In addition, while completing the entire set of movements, the individual was required to suppress interference by irrelevant information and suppress the prepotent responses required for corrective actions, so that the person could respond using top-to-bottom processes that involve harmonious unification and actions related to learning and memory control processes while simultaneously engaging in exercise that frequently involves inhibitory control training. Some studies have shown that the combination of physical and cognitive training may lead to a mutual enhancement of the effects of these two interventions (Hotting and Roder, 2013).

The results of this study also showed that BW exercise did not significantly improve inhibitory control. Previous studies have found that moderate-intensity aerobic exercise can improve an individual’s cognitive function, which is inconsistent with the results of this study (Fox and Raichle, 2007; Song et al., 2020). This might be due to the specific nature of the research subjects, like college students, in this study. There is evidence that the influence of physical activity on cognitive functions is stronger and more obvious in teenagers and the elderly population but weaker in young adults (Voss et al., 2011). Some studies have found that movement complexity plays an important role in cognitive control (Best, 2012). Compared with TCC, BW is a relatively simple and repetitive movement with a single movement form. Our previous research on cognitive flexibility yielded similar results (Cui et al., 2021), suggesting that the improvement of cognitive function in young adults might require movement involving more cognitive operations. On the other hand, this finding may be related to the cycle of exercise, which lasted for 8 weeks in this experiment. In this study, 8 weeks of BW was found to be optimum for modulating spontaneous neural activity, which was reflected in its influence on functional brain activity, but its positive effects have not yet been shown in terms of behavior. Numerically, the inhibitory control RT of the BW group also decreased, but the decrease was not statistically significant. As a result, a longer exercise intervention time might be needed in future studies. Previous behavioral studies have shown that TCC is more beneficial to cognitive function improvement than BW in older adults (Ji et al., 2017). Thus, our study suggests that TCC may improve inhibitory control in young adults and have potential therapeutic advantages.

### Tai Chi Chuan Altered Spontaneous Neural Activity

Our results showed that both TCC and BW intervention could induce spontaneous neural activity, which was consistent with the previous findings (Voss et al., 2011; Best, 2012; Ji et al., 2017; Wei et al., 2017). Both human and animal studies have shown that physical activity promotes neural plasticity in the brain, thus promoting cognitive function (van Uffelen et al., 2008; Smith et al., 2010).

The right paracentral lobule belongs to the key brain area of the motor-sensory network, which innervates motor and sensory modules of the contralateral extremities (Bruchhage et al., 2020; Deng et al., 2021). Throughout an 8-week exercise intervention, the college students gradually transitioned from the initial stage of action acquisition to the stage of action automation. Therefore, TCC decreased the spontaneous activity in the PCL.R, and brisk walking attenuated the enhancement of spontaneous activity in this brain region. The FFG.R involves facial perception (Uono et al., 2017) and body perception (Grewe et al., 2015). Both TCC and BW involve a lot of body perception in the process. For facial perception, the exercise intervention in this study was set in the form of a group, which inevitably involved facial recognition and perception, which might also be one of the reasons for improving its effects. The enhancement of fALFF in the FFG.R by TCC might be caused by the elements of mindfulness and meditation contained in the TCC (Cui et al., 2021). Furthermore, it has been found that TCC can improve levels of mindfulness (Chen et al., 2021). In the process of TCC exercise, individuals need to be guided with their own thoughts and pay more attention to the continuous awareness of movements, breathing, etc., to continuously improve the ability of mindfulness-awareness and make their sensory perception stronger. The SFGdor.R plays an important role in the process of inhibitory control. The dorsolateral prefrontal cortex is part of the executive control network, which is involved in controlling movement and cognitive tasks that require externally directed attention (Beaty et al., 2015). The SFGdor.R is located in the frontal lobe. Previous studies have also shown that functional activity changes in the cerebral cortex after physical exercise training are primarily reflected in the frontal cortex (Wei et al., 2017). Additionally, stress usually has influenced the function of mBDNF and synapse in brain (Nestler et al., 2002; Martinowich et al., 2007; Kojima et al., 2020), the unexpected changes of fALFF in the control group may be induced by participants’ stress reaction during the final examination. This explanation remains speculative, as our study did not measure this pressure, so relevant analysis cannot be carried out, which can be further explored in future research.

Compared to the control group, the participants had four brain areas that displayed improved fALFF after TCC intervention and three brain areas with improved fALFF after the BW intervention. However, there was a significant difference in the SFGmed.L between the TCC and brisk walking groups. The SFGmed.L located in the mPFC, which connects the default mode network and left dorsal attention network, thus playing crucial roles in the ability of impulse control, self-awareness, regulating emotion, and attention (Qiu et al., 2011; Jiang et al., 2016; Park et al., 2018). Several studies have reported that people with schizophrenia (SZ; Cui et al., 2017), autism spectrum disorder (ASD; Yao et al., 2016), and antisocial personality disorder (ASPD; Jiang et al., 2016), and internet gaming disorder (IGD; Park et al., 2018) have abnormal activity in the SFG. TCC involves sequences moving memory and planning, inhibition of incorrect movements, etc. Compared with normal aerobic exercise, TCC practice requires more awareness, attention control, movement control, and memory components that frequently activate a participant’s related functions during exercise. This finding might also be related to the learning process, and a previous study has pointed out that during the acquisition of motor skills, the brain activity shows plasticity (Halsband and Lange, 2006). TCC has more new movement forms that require participants to learn as a new experience. Physical exercise may trigger the process of promoting spontaneous neural activity in the brain, thus improving the ability of individuals to cope with new needs through behavioral adaptations, and the combination of physical training and cognitive training may lead to the mutual reinforcement of the two interventions (Hotting and Roder, 2013). TCC has both characteristics. Therefore, the effect of optimizing spontaneous neural activity in the brain is stronger, which enhances inhibitory control. Another reason might be that TCC had more advantages regarding movement forms, including mindfulness and physical training; hence the difference could be explained by mindfulness. There is a hypothesis that the frontoparietal control network is relevant for meditation and mindfulness skills (Marzetti et al., 2014). A previous review has supported this hypothesis by finding that meditation is related to the left superior temporal gyrus (Tomasino et al., 2014).

Our research showed that TCC also enhanced the fALFF in the SFGmed.L and that the enhancement of fALFF in this brain region could explain the improvements in inhibitory control induced by TCC. Several studies have supported our findings. Yin has shown that 6 weeks of TCC can enhance the ALFF in the left SFG of elderly individuals (Yin et al., 2014). A cross-sectional study has also found a relationship between inhibitory control performance and fALFF among the TCC practitioners (Wei et al., 2017), and two longitudinal studies have indicated that there is a significant change in fALFF after the TCC intervention (Tao et al., 2017b; Mei et al., 2019). But all those studies have focused on older adult populations. Since younger adults are required to use many cognitive resources to solve issues in their day-to-day lives, so it is important to explore the effect of exercise on promoting inhibitory control and spontaneous activity.

This study has several limitations. Firstly, given our small sample size, the findings should be interpreted cautiously. Secondly, the positive effect of BW on inhibitory control was not observed in this study, which might be due to insufficient exercise intervention duration. Larger sample sizes and longer exercise duration studies are warranted to confirm these results in future investigations. Thirdly, we applied a modified flanker task to measure inhibitory control, and the stimulus presentation time was 1,000 ms. However, some studies have used a shorter stimulus presentation time followed by a response window of 1,000–1,500 ms. The inconsistencies in task setting might influence the results of the relationship between inhibitory control performance and functional brain activity. Fourthly, although we comparatively strictly controlled the three groups in the same conditions except for exercise intervention, fALFF changes occurred in some brain regions in the control group. We speculate that this might be caused by the stress of final exams, but the actual causes need to be further explored in the future. Finally, the changes in other brain regions found in this study might be related to other behavioral changes caused by TCC, such as perception, meditation, and mindfulness level. The influence of TCC on these behavioral indicators warrants further analysis.

## Conclusion

In summary, our results indicated that 8 weeks of TCC intervention could improve processing efficiency related to inhibitory control and modulate spontaneous neural activity in young adults, and TCC had potential advantages over BW intervention for optimizing spontaneous neural activity.

## Data Availability Statement

The original contributions presented in the study are included in the article, further inquiries can be directed to the corresponding author/s.

## Ethics Statement

The studies involving human participants were reviewed and approved by National Key Laboratory of Cognitive Neuroscience and Learning. The patients/participants provided their written informed consent to participate in this study.

## Author Contributions

H-CY and LC designed the experiment. LC and Q-QS participated in the exercise intervention, collected and analyzed the data. Q-QS wrote the manuscript with substantial contributions from H-CY and LC. J-YZ, D-LW, YW, and X-JL participated in the exercise intervention. L-NZ edited the manuscript. All authors contributed to the article and approved the submitted version.

## Conflict of Interest

The authors declare that the research was conducted in the absence of any commercial or financial relationships that could be construed as a potential conflict of interest.

## Publisher’s Note

All claims expressed in this article are solely those of the authors and do not necessarily represent those of their affiliated organizations, or those of the publisher, the editors and the reviewers. Any product that may be evaluated in this article, or claim that may be made by its manufacturer, is not guaranteed or endorsed by the publisher.

## Footnotes

^1^ http://www.neurobs.com/

^2^ https://www.nitrc.org/projects/gretna

^3^ https://www.fil.ion.ucl.ac.uk/spm/

^4^ http://restfmri.net/forum/index.php?

^5^ https://www.alivelearn.net/xjview/
